# Pressure and Chemical Unfolding of an α-Helical Bundle Protein: The GH2 Domain of the Protein Adaptor GIPC1

**DOI:** 10.3390/ijms22073597

**Published:** 2021-03-30

**Authors:** Cécile Dubois, Vicente J. Planelles-Herrero, Camille Tillatte-Tripodi, Stéphane Delbecq, Léa Mammri, Elena M. Sirkia, Virginie Ropars, Christian Roumestand, Philippe Barthe

**Affiliations:** 1 Centre de Biologie Structurale INSERM U1054, CNRS UMR 5048, Université de Montpellier, 34090 Montpellier, France; cecile.dubois@cbs.cnrs.fr (C.D.); camille.tillatte@gmail.com (C.T.-T.); stephane.delbecq@umontpellier.fr (S.D.); lea.mammri@gmail.com (L.M.); philippe.barthe@cbs.cnrs.fr (P.B.); 2 Structural Motility, Institut Curie, Paris Université Sciences et Lettres, Sorbonne Université, CNRS UMR144, 75248 Paris, France; vicente@mrc-lmb.cam.ac.uk (V.J.P.-H.); maria-elena.sirkia@curie.fr (E.M.S.); Virginie.ROPARS@cea.fr (V.R.)

**Keywords:** protein folding, NMR, high hydrostatic pressure, thermodynamic stability, α-helical bundle

## Abstract

When combined with NMR spectroscopy, high hydrostatic pressure is an alternative perturbation method used to destabilize globular proteins that has proven to be particularly well suited for exploring the unfolding energy landscape of small single-domain proteins. To date, investigations of the unfolding landscape of all-β or mixed-α/β protein scaffolds are well documented, whereas such data are lacking for all-α protein domains. Here we report the NMR study of the unfolding pathways of GIPC1-GH2, a small α-helical bundle domain made of four antiparallel α-helices. High-pressure perturbation was combined with NMR spectroscopy to unravel the unfolding landscape at three different temperatures. The results were compared to those obtained from classical chemical denaturation. Whatever the perturbation used, the loss of secondary and tertiary contacts within the protein scaffold is almost simultaneous. The unfolding transition appeared very cooperative when using high pressure at high temperature, as was the case for chemical denaturation, whereas it was found more progressive at low temperature, suggesting the existence of a complex folding pathway.

## 1. Introduction

Although small single-domain proteins are generally found to exhibit highly cooperative two-state unfolding transitions [1,2], the thousands of interactions that stabilize their 3D structure are unlikely to form simultaneously and folding intermediates should exist along their folding pathway. Nevertheless, especially in the case of small, fast-folding single-domain proteins, folding intermediates are generally low populated at equilibrium, and cannot be easily identified when using classical thermal or chemical perturbation in association with methods applied to a single probe in the 3D structure of the protein (for instance, intrinsic fluorescence of a tryptophan residue) or with methods giving global structural information (for instance, molar ellipticity in the case of circular dichroism (CD) study).

Multidimensional NMR spectroscopy is a particularly powerful tool to obtain high-resolution structural information about protein folding events because an abundance of site-specific probes can be studied simultaneously in a single spectrum. In the recent past, NMR combined with high-pressure perturbation has emerged as a powerful tool to explore in detail the folding landscape of small proteins, at a quasi-atomic resolution [3,4,5,6]. Indeed, contrary to chemical or thermal denaturation, which acts globally and depends on exposed surface area in the unfolded state, pressure denaturation depends on the elimination of the solvent-excluded internal voids, due to imperfect protein packing, by water penetration inside the core of the protein [7,8,9]. Thus, because the distribution of solvent-excluded voids depends on the protein structure, the pressure-induced unfolding originates from unique properties of the folded state.

High-pressure NMR unfolding studies have been applied to several single-domain proteins in order to characterize their folding landscape. Until now, these studies have concerned essentially all-β [10,11] or mixed-α/β [9,12,13] protein scaffolds, and similar studies are lacking for all-α structures although they represent a widespread assembly motif [14]. In the present manuscript, we report the NMR study of the folding of an α-helical bundle, the GH2 domain of the protein adaptor GIPC1 [15]. GIPC is an adaptor protein that binds and regulates vesicular trafficking of many transmembrane proteins [15]. The X-ray structure of GIPC1 has been solved [16] and shows that the full-length protein exists as a dimer in the crystal (Supplementary Materials, Figure S1). Each monomer contains three well-identified domains: a central PDZ domain flanked by an N-terminal GIPC-homology 1 (GH1) domain and a C-terminal GH2 domain [16]. The structure of the PDZ domain displays a typical PDZ fold with five β-strands and two α-helices. The GH1 domain adopts a ubiquitin-like fold composed of four β-strands and one α-helix, and the GH2 domain forms a four-helix globular fold. After solving the solution structure of the GIPC1-GH2 domain, we studied its folding/unfolding pathway at a residue-specific level using 2D NMR spectroscopy combined with high-pressure perturbation at three different temperatures, and with chemical perturbation. As a result, we found that whereas high-pressure NMR reveals the existence of a partial unfolding at low temperature, unfolding becomes highly cooperative at higher temperature or when using chemical perturbation.

## 2. Results

### 2.1. NMR Resonance Assignments and Solution Structure of GIPC1-GH2

**Figure 1:**
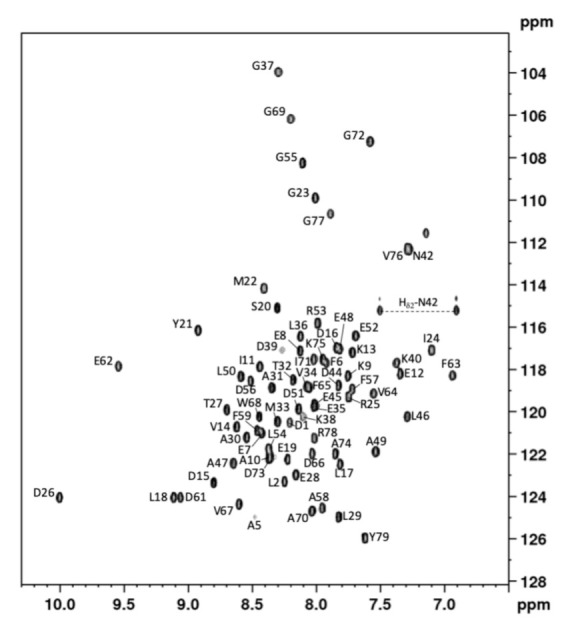
GIPC1-GH2 NMR fingerprint. [^1^H-^15^N] HSQC spectrum of GIPC1-GH2 at 800 MHz, 20 °C on a 0.5 mM, ^15^N uniformly labeled sample dissolved in a 20 mM Tris-HCl pH 7.2, 150 mM NaCl buffer. Cross-peak assignments are indicated using the one-letter amino acid and number code.

Proton, nitrogen, and carbon NMR resonances of GIPC1-GH2, renumbered 1–79 for simplicity, have been assigned and its solution structure solved using essentially [^1^H,^15^N,^13^C] triple-resonance and [^1^H,^15^N] double-resonance 3D NMR spectroscopy (see Section 4) with the classical sequential assignment strategy. ^1^H and ^15^N resonances have been assigned for all amide groups of nonproline (76) residues (Figure 1), and Cα, Cβ, C’ resonances for 96.2% residues. Resonance assignments have been deposited at the BMRB data bank (BMRB code 34609).

NOEs were measured on a 3D [^1^H,^15^N] NOESY-HSQC experiment and on a 2D [^1^H,^1^H] NOESY spectrum recorded in a deuterated buffer. Dihedral restraints (φ, ϕ, and χ_1_) were obtained from TALOS-N [17] analysis of backbone atom chemical shifts. After conversion into distance restraints, these data sets were used with CYANA [18] to build the 3D structure of GIPC1-GH2. H-bond restraints were also used for the structure modeling. Usually, these restraints are deduced from hydrogen/deuteron (H/D) exchange experiments, yielding amide protons potentially involved in H-bonds as donor atoms. In the case of GIPC1-GH2, H/D exchange rates were unusually fast, even at low temperature (5 °C): all amide proton resonances disappeared during the few minutes needed for the setting of the experiments, preventing the use of this approach. Instead, we used CLEANEX-PM [19,20] experiments to determine which amide protons are solvent-exposed in the 3D structure. In these experiments, the water resonance was selectively excited, and water magnetization transferred to solvent-exposed amide protons with an appropriate spin-locking module. This sequence was incorporated in a conventional HSQC scheme to resolve amide peaks along the ^15^N indirect dimension. Thus, amide corresponding cross-peaks which were not present in this experiment (Supplementary Materials, Figure S2) and which belonged to residues exhibiting φ, ψ values characteristic of secondary structure elements (α-helices, in the present case), as determined from TALOS analysis, were considered as involved in a regular H-bond, and the corresponding distance restraints were used for structure modeling.

**Figure 2:**
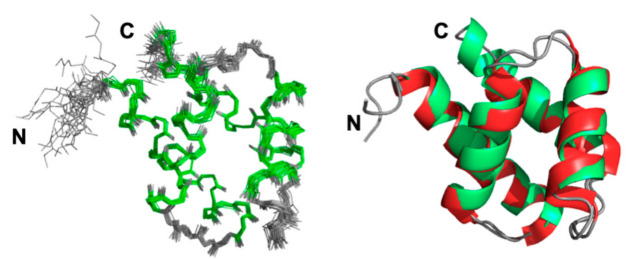
Solution structure of GIPC1-GH2. (**Left**) Overlay of the 20 best NMR structures (backbone atoms only) with lowest energy. The regular α-helices are colored in green. (**Right**) Superimposition of ribbon representations of the solution structure (with α-helices in green and the X-ray structure with α-helices in red) of the GH2 domain extracted from the structure of the full-length protein (PDB code: 5V6b).

A final set of 1219 restraints was used, and the pairwise rmsd calculated for backbone heavy atoms between the 20 best refined structures was 0.47 Å (residues 5–76) (Figure 2). Most of the residues (95.6%) fall in the most favored region of the Ramachandran plot, with no residue in the generously allowed or disallowed regions, highlighting the high quality of our model (see structural statistics, Supplementary Materials, Table S1). The solution structure of GIPC1-GH2 consists of four antiparallel amphipathic helices arranged in an α-helical bundle. It is virtually identical to the X-ray structure adopted by this domain in the full-length protein, as shown by their superimposition displayed in Figure 2. An rmsd value of 0.85 Å was measured between them for all backbone heavy atoms (residues 5–73). This value drops to 0.68 Å when considering only backbone heavy atoms involved in helices. The bundle can be divided in two subdomains, each one consisting of two antiparallel helices stapled against each other: the N- (helix I) and C-terminal (helix IV) helices form the first subdomain, while the α-hairpin made by helix II and III forms the second subdomain. These two subdomains make an angle of approximately 50° in the 3D structure of GIPC1-GH2. The structure coordinates of the NMR structure of GIPC1-GH2 have been deposited at the Protein Data Bank (PDB code 7NRN).

**Figure 3:**
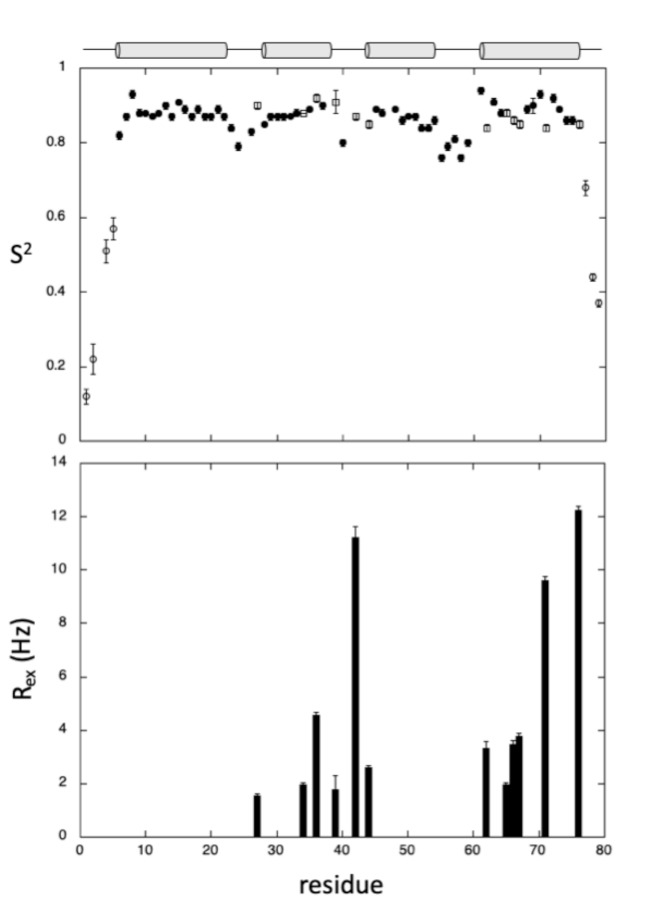
Intrinsic dynamics of GIPC1-GH2. (**Top**) Generalized order parameters *S*^2^ obtained from Lipari–Szabo analysis plotted versus the protein sequence. The regular two-parameter spectral density function (Section 4, Equation (1)) has been used for residues plotted as filled circles, whereas the extended Lipari–Szabo formalism (Section 4, Equation (3)) has been used for residues plotted as open circles. Open squares correspond to residues for which *J*(0) values have been corrected from exchange contributions (Section 4, Equation (2)). (**Bottom**) R_ex_ contributions obtained from this last equation are reported versus the sequence for residues exhibiting conformational exchange. The location of the four helices in the protein sequence is schematized with cylinders on top of the figure.

The intrinsic dynamics of GIPC1-GH2 were also investigated by measuring heteronuclear ^15^N T_1_, T_2_ relaxation times and [^1^H,^15^N] heteronuclear nOes, and converting these parameters into *J*(0), *J*(ω_N_), and <*J*((ω_H_)> spectral densities through Solomon equations [21] (see Section 4 and Supplementary Materials, Figure S3). Spectral densities were then fitted with model-free Lipari–Szabo equations [22] to extract the global (*τ_c_*) and internal (*τ_e_*) correlation times of the molecule, and generalized order parameters *S*^2^ for each residue (Figure 3). A *τ_c_* value of 6.09 ns was obtained from the fit of the relaxation parameters, in good agreement with the expected correlation time of this small protein at 20 °C.

The regular Lipari–Szabo model was used for most of the residues involved in α-helices, yielding *S*^2^ values close to 0.85, confirming that these secondary structure elements are well defined. The extended Lipari–Szabo model [23] was needed to fit the N- and C-terminal residues, suggesting the existence of more complex motions in these flexible regions, as usually observed. Interestingly, adding exchange contribution (R_ex_) to *J*(0) was mandatory to fit some residues located in the loops connecting the four helices, but also in helix IV and, to a lesser extent, helix II, suggesting that these two helices are prone to conformational exchange.

### 2.2. GIPC1-GH2 Denaturation Studies

#### 2.2.1. Pressure Denaturation

**Figure 4:**
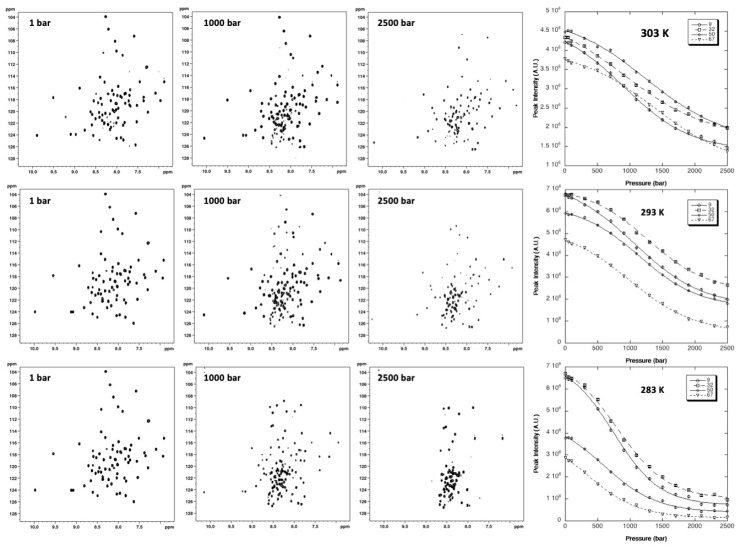
NMR detected high-pressure unfolding of GIPC1-GH2 at 30 °C, 20 °C, and 10 °C (from top to bottom). At each temperature, examples of [^1^H,^15^N] HSQC at 1, 1000, and 2500 bar are displayed from left to right. The rightmost panels report overlays of four (residues K9, T32, L50, and V67) residue-specific experimental denaturation curves obtained from the fits of the pressure-dependent sigmoidal decrease of the corresponding residue cross-peak intensities in the HSQC spectra with Equation (4).

2D [^1^H,^15^N] HSQC spectra of ^15^N uniformly labeled GIPC1-GH2 were recorded at variable pressures (1 to 2500 bar) and at 10, 20, and 30 °C (Figure 4). As usually found, the intensity of each native state peak decreases as a function of pressure, while the intensity of peaks corresponding to the unfolded state, centered around 8.5 ppm in the proton dimension, increases concomitantly. This supports a slow equilibrium on the NMR timescale for each residue between the native and unfolded state, and a two-state transition for each residue between their native/unfolded states during the unfolding process. Thus, even though the global protein unfolding does not likely conform to a two-state transition, locally this simple model can be used to interpret the loss of intensity for each native state cross-peak [6].

**Figure 5:**
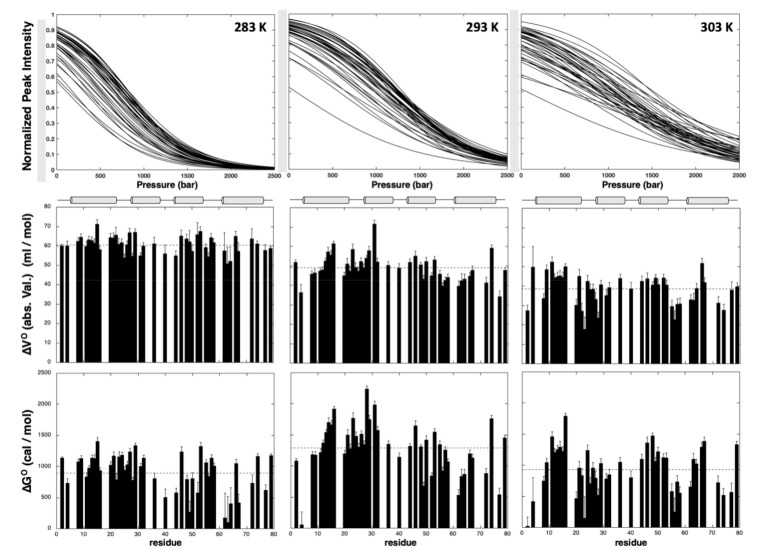
Steady-state thermodynamic parameters measured for GIPC1-GH2 at 10 °C, 20 °C, and 30 °C (from left to right) from residue-specific pressure denaturation curves. From top to bottom: overlay of the normalized residue-specific denaturation curves as obtained from the fit of the pressure-dependent sigmoidal decrease of the residue cross-peak intensities in the HSQC spectra with Equation (4); residue-specific values (absolute values) of the apparent volume change of unfolding $\Delta V_{u}^{0}$ plotted versus the protein sequence; residue-specific values of the apparent free energy of unfolding $\Delta G_{u}^{0}$ plotted versus the protein sequence. The dashed lines represent the mean values of the measured thermodynamic parameters. The location of the four helices in the protein sequence is schematized with cylinders on top of the graphics.

A total of 44 residues (58% of the nonproline residues) gave overlapping cross-peaks neither in the folded state nor in between the folded and unfolded states at any of the temperatures used for the study. These residues displayed cross-peaks of sufficient intensity at atmospheric pressure to be accurately fitted to the two-state pressure-induced unfolding model described in the Section 4 (Equation (4)), Figure 4, giving a substantial number of local probes for the description of the GIPC1-GH2 folding pathway. At the residue level, the two-state model was adequate to fit all individual unfolding curves, but yielded significantly different values for apparent free energy $\Delta G_{u}^{0}$ and apparent volume change $\Delta V_{u}^{0}$ (Figure 5) of unfolding, suggesting a substantial deviation from a two-state behavior for the global unfolding of the protein, whatever the temperature of the study. The asymmetric distributions observed for apparent $\Delta G_{u}^{0}$ and apparent $\Delta V_{u}^{0}$ strongly support this assumption and suggest that partial unfolding of the molecule should appear when increasing the pressure (Supplementary Materials, Figure S4).

GIPC1-GH2 displayed a weak stability that appeared to be maximum at 20 °C, with an average value for the apparent free energy of unfolding <$\Delta G_{u}^{0}$> of 1293 ± 62 cal/mol, and significantly decreased at higher (<$\Delta G_{u}^{0}$> = 925 ± 57 cal/mol at 30 °C) or lower ((<$\Delta G_{u}^{0}$> = 903 ± 49 cal/mol at 10 °C) temperatures. Also, a linear decrease with temperature was observed for the average values (in magnitude mode) of apparent $\Delta V_{u}^{0}$ (<$\Delta V_{u}^{0}$> = −61 ± 4 mL/mol, −49 ± 7 mL/mol, and −38 ± 8 mL/mol at 10, 20, and 30 °C, respectively). The temperature-dependent decrease in $\Delta V_{u}^{0}$ is a well-known effect due to the difference in thermal expansion between the folded and unfolded states [24].

Average values of $\Delta G_{u}^{0}$ calculated for each helix indicate differences in local stability (Supplementary Materials, Figure S5). Interestingly, this local stability depends on the temperature. At 10 °C and 20 °C, helix I and II appear slightly more stable than helix III and IV, helix II being the most stable at 20 °C whereas it has a similar stability as helix I at 10 °C. At 30 °C, the highest values of $\Delta G_{u}^{0}$ are found in helix I and III. Importantly, the local thermal stability measured for each helix follows the thermal stability of the whole domain, with a maximum observed at 20 °C. Likewise, a similar decrease with temperature is observed for average $\Delta V_{u}^{0}$ values calculated for each helix and for the average value calculated over the whole structure, but without any significant variations between the four helices.

Information brought by normalized residue-specific denaturation curves has been used to track and to characterize possible intermediates in the folding pathway of GIPC1-GH2 [6,8]. Thus, at a given pressure, the value of 1 measured for a given cross-peak (*I* = *I_f_* = 1; Equation (4)) is associated with a probability $P_{i}$ of 1 (100%) to find the corresponding residue “*i*” in the native state, while a residue “*j*” for which the corresponding cross-peak has disappeared (*I* = *I_U_* = 0; Equation (4)) from the HSQC spectrum has a probability $P_{j}$ equal to zero to be in a native state. Since these probabilities are related to the “native fraction” for a given residue, they are called fractional probabilities.

**Figure 6:**
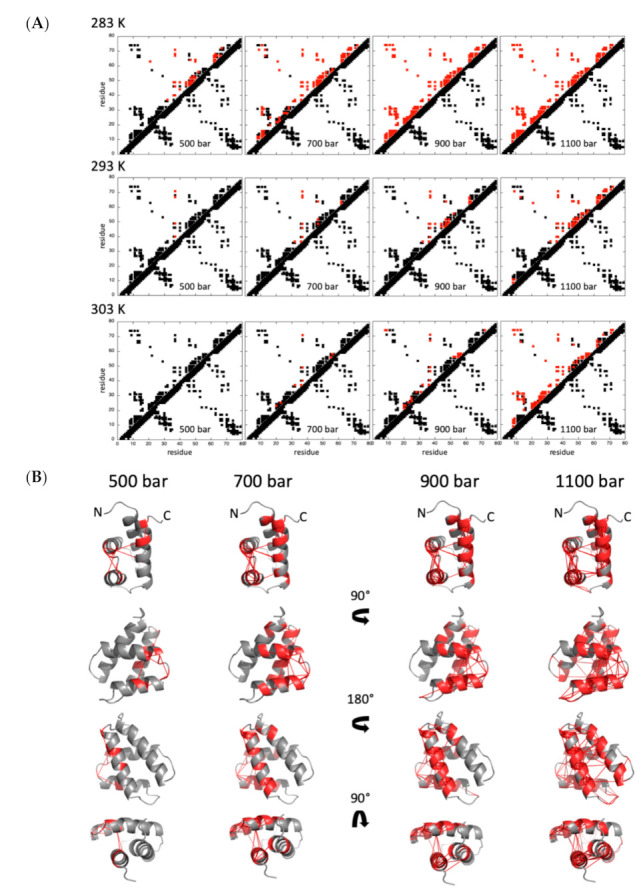
Pressure denaturation of GIPC1-GH2. (**A**) Contact maps built from the best solution structure obtained for GIPC1-GH2 at 500, 700, 900, and 1100 bar, and at 283, 293, and 303 K, as indicated. Contacts below the diagonal have been calculated with CMview (http://www.bioinformatics.org/cmview/; accessed on 25 March 2020): they correspond to residue where the distance to the corresponding Cα is lower than 9 Å. Above the diagonal, only the contacts for which fractional probability can be obtained have been reported. In addition, contacts have been colored in red when contact probabilities P_ij_ lower than 0.5 are observed. (**B**) Visualization of the probabilities of contact on ribbon representations of GIPC1-GH2 at 20 °C and at 500, 700, 900, and 1100 bar, as indicated. The red lines represent contacts that are significantly weakened (P_ij_ ≤ 0.5) at the indicated pressure. Residues involved in these contacts are also colored in red. The arrows in the middle of the panel indicate the rotation between the different views.

Given a pressure where these two residues *i* and *j* are in an intermediate situation (0 < $P_{i}$ and $P_{j}$ < 1), and if these two residues are in contact in the native state (at atmospheric pressure), their fractional probability P_ij_ to be in contact at this pressure is given by the geometric mean of the two individual probabilities: P_ij_ = $\sqrt{P_{i}\times P_{j}}$ [12] (Figure 6).

At 20 °C, the temperature where GIPC1-GH2 exhibits the highest stability, the pressure dependence of the contact maps shows that helix III and IV are the first regions affected by an increase of pressure: tertiary contacts between these two helices are already significantly weakened at 500 bar (P_ij_ ≤ 50%), as well as secondary contacts characteristic of the helical structure (Figure 6). This partial unfolding concerns mainly these two helices up to 900 bar. Above this pressure, we observed a loss of contacts between helix IV and helix I, while helix I and II remain unaffected until 1100 bar. The unfolding of these two helices, as well as the loss of tertiary contacts between them, is observed at higher pressure (1300 bar, not shown). An identical scenario is observed at 10 °C, but with a shift to lower pressures, consistent with the lower stability of the protein at this temperature. At 700 bar, helix III and IV are unfolded (P_ij_ ≤ 50%), and local unfolding already concerns helix I and II, while all the contacts are lost at 900 bar. While a similar stability of the protein is observed at 30 °C, a rather different scenario is observed for unfolding. The structure remains stable until 900 bar, with little loss of tertiary contacts observed. At 1100 bar, a sharp unfolding transition is observed, that concerns both the tertiary contacts between the four helices and the secondary contacts in all the helices, simultaneously. Finally, we observed a global unfolding of the molecule at 1300 bar.

#### 2.2.2. Chemical Denaturation

2D [^1^H,^15^N] HSQC spectra of ^15^N uniformly labeled GIPC1-GH2 were recorded at 20 °C, the temperature where the protein exhibits the highest stability, and at increasing urea concentrations.

**Figure 7:**
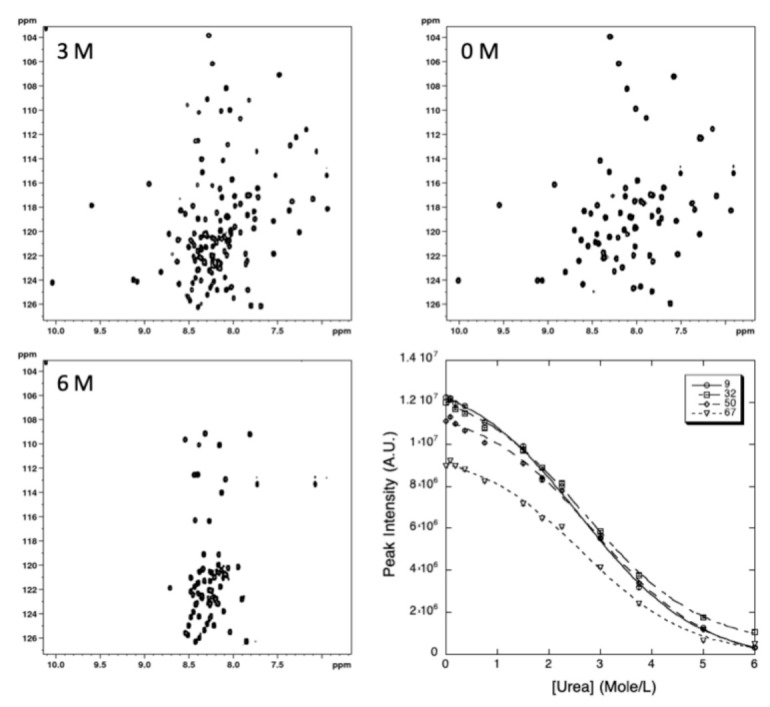
NMR detected chemical unfolding of GIPC1-GH2 at 20 °C. Examples of [^1^H,^15^N] HSQC at 0, 3, and 6 M urea are displayed. The last panel shows an overlay of four (residues K9, T32, L50, and V67) residue-specific denaturation curves obtained from the fits of the urea concentration-dependent sigmoidal decrease of the corresponding residue cross-peak intensities in the HSQC spectra with Equation (5).

**Figure 8:**
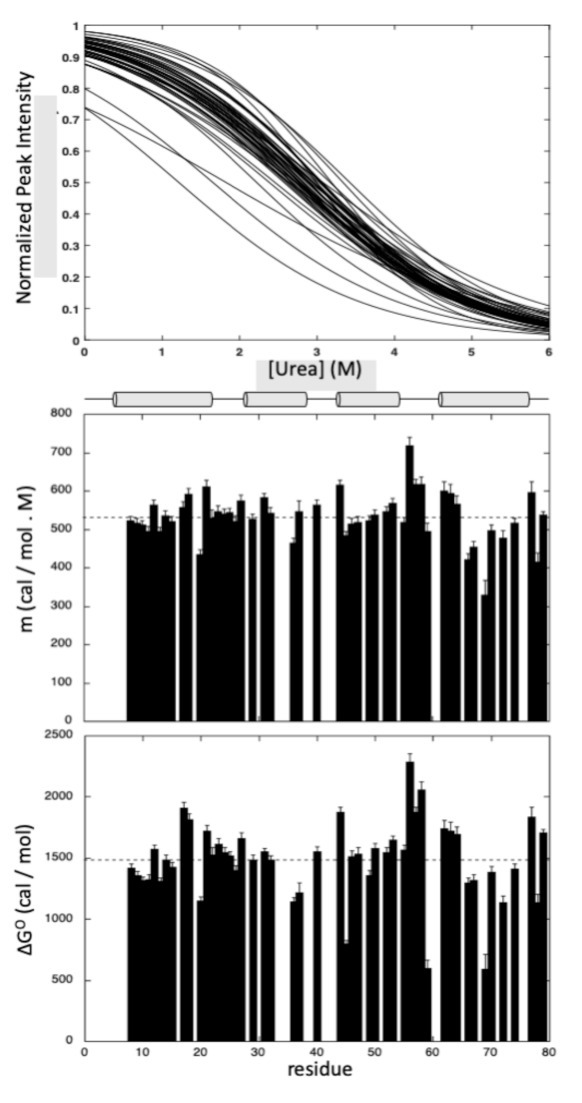
Steady-state thermodynamic parameters measured for GIPC1-GH2 at 20 °C from residue-specific urea denaturation curves. From top to bottom: overlay of the normalized residue-specific denaturation curves as obtained from the fit of the urea concentration-dependent sigmoidal decrease of the residue cross-peak intensities in the HSQC spectra with Equation (5); residue-specific values of the apparent m-values plotted versus the protein sequence; residue-specific values of the apparent free energy of unfolding $\Delta G_{u}^{0}$ plotted versus the protein sequence. The dashed lines represent the mean values of the measured thermodynamic parameters. The location of the four helices in the protein sequence is schematized with cylinders on top of the graphics.

A total of 49 residues (64% of the nonproline residues) gave overlapping cross-peaks neither in the folded state nor in between the folded and unfolded states, and these residues can be accurately fitted to the two-state pressure-induced unfolding model described in the Section 4 (Equation (5), Figure 7). As observed for pressure denaturation, the intensity of each native state peak decreases as a function of urea concentration in the NMR sample, while the intensity of peaks corresponding to the unfolded state increases concomitantly, supporting a slow equilibrium on the NMR timescale for each residue between the native and unfolded state during the unfolding process. As reported above for pressure denaturation, a two-state transition model has been used to interpret the loss of intensity for each native state cross-peak (see Section 4). The residue-specific values obtained for the apparent free energy $\Delta G_{u}^{0}$ of unfolding and for the apparent m-values are displayed in Figure 8.

As previously observed, GIPC1-GH2 displays a weak stability at 20 °C, with an average free energy value for unfolding <$\Delta G_{u}^{0}$> of 1484 ± 45 cal/mol, a value close to that measured from pressure denaturation curves at the same temperature. But contrary to what we observed with pressure denaturation, we did not observe significant variations in between the different helices when looking at the average values of $\Delta G_{u}^{0}$ calculated for each helix (Supplementary Materials, Figure S6).

**Figure 9:**
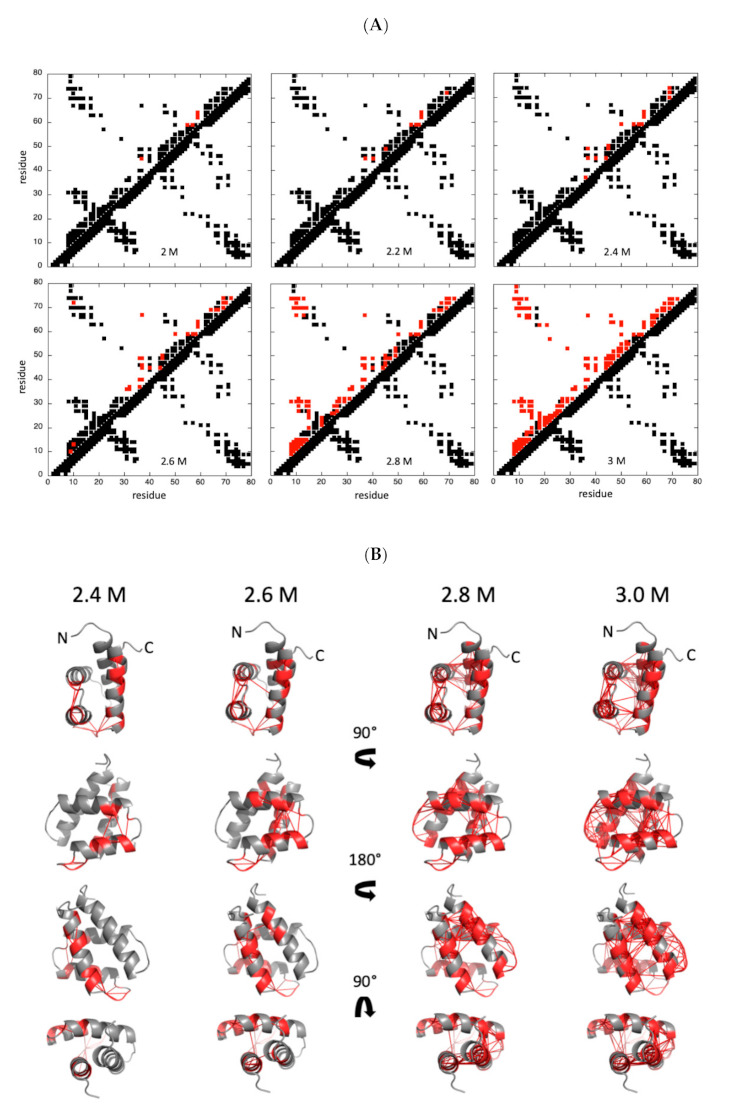
Chemical denaturation of GIPC1-GH2. (**A**) Contact maps built from the best solution structure obtained for GIPC1-GH2 at 20 °C, and at 2, 2.2, 2.4, 2.6, 2.8, and 3 M urea, as indicated. Contacts below and above the diagonal are displayed following the same rules as in Figure 6. (**B**) Visualization of the probabilities of contact on ribbon representations of GIPC1-GH2 at 20 °C and at 2.4, 2.6, 2.8, and 3 M urea, as indicated. The red lines represent contacts that are significantly weakened (P_ij_ ≤ 0.5) at the indicated pressure. Residues involved in these contacts are also colored in red. The arrows in the middle of the panel indicate the rotation between the different views.

As in the case of pressure denaturation, we built fractional contact maps from probabilities of contact calculated from fractional probabilities of individual residues extracted from the normalized residue-specific chemical denaturation curves obtained at 20 °C (Figure 9).

Interestingly, if some contacts are lost between the C-terminal end of helix IV and the N-terminal end of helix III at low urea concentrations ([urea] ≤ 2.4–2.6 M), a sharp unfolding transition takes place between 2.6 and 2.8 M urea that concerns both secondary and tertiary contacts in the four helices. In this view, the folding scenario looks like what was observed for pressure denaturation at 30 °C, where an increased unfolding cooperativity was observed when compared to 10 °C or 20 °C.

## 3. Discussion

The structure in solution of the GH2 domain of GIPC1 shows that this domain keeps its α-helical bundle fold outside the full-length protein context, even though it exhibits a rather low stability, probably due to the loss of intra- and intermolecular interactions that occur within the dimeric structure of the full-length protein. Indeed, this low stability is supported by the fast exchange rates exhibited by the amide protons that cannot be measured by regular H/D exchange NMR experiments. Nevertheless, CLEANEX-PM experiments showed that the amides involved in the H-bonds stabilizing the helical structures are not solvent-exposed, contrary to those located in the loops linking the different helices and in the flexible N- and C-terminal ends of the domain, supporting the idea that they are involved in the regular H-bonds expected in those regular elements of secondary structure. In addition, *S*^2^ values close to 1, as obtained from the ^15^N relaxation study, indicate that the helices are well structured, even though significant exchange contributions ($R_{2}^{ex}$) can be observed, especially in helix IV. This suggests that this helix is prone to conformational exchange. Notably, GIPC1-GH2 was found to be stable only in a limited range of temperature. The stability was found to be maximum at 20 °C, whereas it decreases significantly when increasing or decreasing the temperature. As evaluated from the ratio of the intensity of native/unfolded amide cross-peaks of representative residues measured on HSQC spectra, the fraction of native protein is about 70% at 40 °C and 60% at 0 °C (Supplementary Materials, Figure S7), indicating that the protein is sensitive both to thermal and to cold denaturation. GIPC1-GH2 is also very sensitive to high hydrostatic pressure, since it unfolds at 20 °C in the 1–2500 bar range without adding any sub-denaturant concentration of chaotropic reagents, as was usually observed for high-pressure denaturation of all-β or mixed-α/β structures in our previous study: sub-denaturant concentration ranging from 0.5 M [25] to about 2 M [8,10] of guanidinium chloride was used to tune the stability of these proteins into the pressure range allowed by the experimental set-up.

Looking closely to the unfolding pathways of GIPC1-GH2 under high hydrostatic pressure, we observed unexpected results. Indeed, since high-pressure denaturation is closely related to the presence of dehydrated internal solvent-excluded voids in the structure, we expected a two-step process starting with the loss of tertiary contacts within the 3D structure, followed by the loss of the secondary contacts yielding helices unfolding. This is because helices are well-packed secondary structure elements, without significant voids inside, while packing defaults are expected within the tertiary structure of the α-helical bundle. In fact, whatever the temperature of the study, local or global unfolding entails the quasi-simultaneous loss of the tertiary and secondary contacts in the concerned areas, meaning that helices are not stable outside the 3D scaffold context. Depending on the temperature used for the high-pressure denaturation study, we observed significant differences in the unfolding process. At 20 °C, the temperature where GIPC1-GH2 exhibits the highest stability, a partial unfolding of the molecule occurs first at helix III and helix IV, while helix I and helix II remain intact until approximately 1300 bar (note that for clarity of the discussion, we consider that a contact between two residues i and j is lost when P_ij_ ≤ 50%). The same scenario is observed at 10 °C, shifted at lower pressure: unfolding of helix III and IV starts below 500 bar instead of 900 bar at 20 °C, while helix I and II start to unfold at 700 bar. A different scenario is observed at 30 °C, where the four helices unfold almost simultaneously, with the onset of unfolding around 1000 bar. Interestingly, globally the protein appears to be more stable at high temperature (30 °C) than at low temperature: at 10 °C, the protein appears completely unfolded (P_ij_ ≤ 50%) at 900 bar while some residual structures are still present at 1100 bar and 30 °C.

This scenario described for high pressure denaturation at high temperature is very similar to what is observed for chemical denaturation of GIPC1-GH2 at 20 °C. We did not observe significant partial unfolding of any helix upon increase in urea concentration but rather a global unfolding of the molecule between 2.6 and 2.8 M urea. This is probably due to the different rules underlying high-pressure and chemical unfolding. As reported above, pressure unfolding is linked to the presence of solvent-excluded voids inside the 3D structure of the protein, and hence depends on the structure of the native state of the protein. On the contrary, the chemical denaturation process is driven by the increase of solvent-accessible area of the unfolded state with respect to the folded state, and is more dependent on the size of the protein [26,27,28]. This probably explains the difference that we observed in the folding pathway between high-pressure denaturation of GIPC1-GH2 at 20 °C or 10 °C, and its chemical denaturation at 20 °C. Note that thermal denaturation is also related to the solvent-accessible area of the unfolded state: this could explain the similar scenario observed for high-pressure denaturation at high temperature (30 °C) and for chemical denaturation. Indeed, at 30 °C, the stability of GIPC1-GH2 is decreased, and thermal denaturation probably competes with high-pressure denaturation, sweeping away the partial unfolding occurring at lower temperature.

## 4. Materials and Methods

### 4.1. Protein Expression and Purification

The construct GIPC1-GH2 domain (residues 255–333) was subcloned in pProEXHTB, allowing the expression of a 6xHis-TEV fusion protein, and was transformed into E. coli BL21-Gold (DE3) (Stratagene, Amsterdam, The Netherlands). Uniform ^15^N or ^15^N/^13^C labeling was obtained by growing cells in minimal M9 medium containing ^15^NH4Cl and/or ^15^NH4Cl/^13^C-u-labeled glucose as the sole nitrogen or carbon sources (Cortecnet). Protein was expressed overnight at 20 °C after induction with 0.2 mM IPTG. Cells were collected by centrifugation and suspended in lysis buffer comprising 20 mM Tris-HCl buffered at pH 7.5 and containing 150 mM NaCl, 2 mM imidazole, and a cOmplete™ EDTA-free tablet (Roche). Cells were lysed by sonication (1 s bursts for 4 min, at 30% amplitude with a large probe, Branson). Cell debris and insoluble materials were removed by centrifugation (Beckman Coulter Avanti J-20 XP centrifuge equipped with a 25.50 rotor, set at 20,000 rpm, at 6 °C). The supernatant was loaded through a benchtop peristaltic pump (Cytiva) onto a cOmplete™ His-Tag Purification Column (Roche, Basel, Switzerland) equilibrated with lysis buffer. After elution with lysis buffer supplemented with 200 mM imidazole, fractions containing the protein were dialyzed with homemade recombinant His tagged rTEV protease (mixed at 100:1 ratio) overnight at 4 °C in 20 mM Tris-HCl buffered at pH 7.5, 150 mM NaCl, and 1 mM imidazole. Cleavage was checked with SDS-PAGE and loaded again into a cOmplete™ His-Tag Purification Column equilibrated with the same buffer used for dialysis in order to remove the protease and the cleaved 6xHis tag. The GipC1-GH2 domain was finally injected through an AKTA system into a Superdex S75 16/60 (GE Healthcare) column, equilibrated with 20 mM Tris-HCl buffered at pH 7.2, 150 mM NaCl. The fractions containing the pure protein were pooled, concentrated to about 1 mM (protein concentration) (Vivaspin 15R, Sartorius). PMSF and EDTA were added (0.1 mM) to the samples that were then flash-frozen in liquid N2 and stored at −80 °C until NMR analysis.

### 4.2. NMR Assignments and Solution Structure

Protein samples were dissolved in 200 µL of aqueous buffer containing 20 mM Tris-HCl pH 7.2, 150 mM NaCl, and 0.1 mM PMSF and EDTA (5% D_2_O for the lock) at a concentration of about 1 mM. Experiments were recorded at 20 °C on a Bruker AVANCE III 800 MHz (Bruker Biospin, Wissenbourg, France) equipped with a 5 mm Z-gradient TCI cryogenic probe head. ^1^H chemical shifts were directly referenced to the methyl resonance of DSS, while ^13^C and ^15^N chemical shifts were referenced indirectly to the ^13^C/^1^H and ^15^N/^1^H absolute frequency ratios. All NMR experiments were processed with Gifa [29].

Backbone and Cβ resonance assignments were made using standard 3D triple-resonance HNCA, HNCACB, CBCA(CO)NH, HNCO, and HN(CA)CO experiments [30] and 3D [^1^H,^15^N] NOESY-HSQC (mixing time 150 ms) and TOCSY-HSQC (isotropic mixing: 60 ms) experiments performed on the ^15^N,^13^C-labeled GIPC1-GH2 sample. [^1^H,^15^N] NOESY-HSQC was used to extract the set of nOe’s restraints used for structure modeling, completed by restraints obtained from a 2D homonuclear NOESY (mixing time 200 ms) recorded on a deuterated buffer. NOE cross-peaks were assigned through automated NMR structure calculations with CYANA 3 [18]. Backbone φ, ψ, and side-chain χ^1^ torsion angle constraints were obtained from a database search procedure on the basis of backbone (^15^N, HN, ^13^C’, ^13^Cα, Hα, ^13^Cβ) chemical shifts using TALOS-N [17]. Hydrogen bond restraints were derived from the analysis of residue (φ,ψ) values and CLEANEX-PM experiments [19,20]. When identified, the hydrogen bond was enforced using the following restraints: ranges of 1.8–2.0 Å for d(N-H,O), and 2.7–3.0 Å for d(N,O).

The final list of restraints, from which values that were redundant with the covalent geometry were eliminated, was used for structure modeling. A total of 200 three-dimensional structures were generated using the torsion angle dynamics protocol of CYANA 3 from 1219 NOEs, 88 hydrogen bonds, and 164 angular restraints. The 20 best structures (based on the final target penalty function values) were minimized with CNS 1.2 according to the RECOORD procedure [31] and analyzed with PROCHECK [32]. The rmsds were calculated with MOLMOL [33]. Models are displayed with PyMOL [34]. All statistics are given in Supplementary Materials, Table S1.

### 4.3. Relaxation Studies

Relaxation rate constant measurements were performed on a 1 mM ^15^N-labeled protein sample, at 14.1 T (600 MHz), using a Bruker AVANCE III spectrometer equipped with a 5 mm Z-gradient TXI probe head. The pulse sequences used to determine heteronuclear ^15^N R_1_, R_2_ relaxation rates, and ^15^N{^1^H}NOE values were similar to those described [35,36,37], and experimental parameters and processing were previously reported in detail for other proteins studied in the laboratory [38,39,40]. The ^15^N longitudinal relaxation rates R_1_ were obtained from nine standard inversion-recovery experiments, with relaxation delays ranging from 18 ms to 1206 ms. The ^15^N transverse relaxation rates R_2_ were obtained from eight standard CPMG experiments, with relaxation delays ranging from 16 ms to 128 ms. Heteronuclear ^15^N{^1^H} NOE were determined from the ratio of two experiments, with and without saturation.

Relaxation data analysis: *J*(0), *J*(ω_N_), and <*J*((ω_H_)> spectral densities were calculated from ^15^N heteronuclear R_2_, R_1_, and ^15^N{^1^H} NOE using the so-called reduced spectral density mapping [35,36,41,42,43,44].

The model-free approach of Lipari and Szabo [22] was then used to further describe the mobility in terms of specific types of motion. This formalism makes the assumption that overall and internal motions contribute independently to the reorientation time correlation function of ^15^N-^1^H vectors and that internal motions occur on a much faster time scale than the global rotation of the molecule. For a protein with isotropic tumbling protein, one obtains:

(1) $$J\left(\omega \right)=\frac{2}{5}\left\{S^{2}\frac{\tau_{c}}{1+{\left(\omega \tau_{c}\right)}^{2}}+\left(1-S^{2}\right)\frac{\tau }{1+{\left(\omega \tau \right)}^{2}}\right\}$$

where *τ* is the harmonics of the overall and the internal (fast) correlation time which pertains to each residue: $\tau^{-1}=\tau_{c}^{-1}+\tau_{f}^{-1}$. Fast internal motions are characterized by the square of a generalized order parameter *S*^2^, which describes the relative amplitude of internal motions and ranges from 0 to 1, and an internal correlation time $\tau_{f}^{}$ for the internal motions.

For some of the residues, the simple form of equation (1) turns out to be insufficient to fit the whole set of experimental data. This occurs for residues where observed *J*(0) values are higher than expected, due to exchange contributions $R_{2}^{ex}$. In this case, the expression for the observed spectral density at 0 frequency is:

(2) $$J\left(0\right)=\frac{2}{5}\left\{S^{2}\tau_{c}+\left(1-S^{2}\right)\tau \right\}+\;\lambda R_{2}^{ex}]$$

where *λ* is a scale factor. This occurs also when residues exhibit internal motions in a time window close to 1 ns. In this case, the expression for the spectral density function is extended to [23]:

(3) $$J\left(\omega \right)=\frac{2}{5}\left\{S_{f}^{2}S_{s}^{2}\frac{\tau_{c}}{1+{\left(\omega \tau_{c}\right)}^{2}}+S_{f}^{2}\left(1-S_{s}^{2}\right)\frac{\tau }{1+{\left(\omega \tau \right)}^{2}}\right\}$$

with $\tau^{-1}=\tau_{c}^{-1}+\tau_{s}^{-1}$, where $S_{f}^{2}$ and $S_{s}^{2}$ are the square of the partial order parameters for fast (picosecond time scale) and slow ($\tau_{s}^{}$, nanosecond time scale) internal motions, respectively. The square of the generalized order parameter *S*^2^, defined as $S_{f}^{2}S_{s}^{2}$, is a measure of the total amplitude of the internal motions. Note that *S*^2^ equals $S_{f}^{2}$ in Equations (1) and (2). Equation (3) assumes that the contribution of the fastest motion to the spectral density function is negligible.

The values of the motional parameters of the individual residues can be derived from the fit of experimental *J*(0), *J*(60 MHz), and <*J*(600 MHz)> using Equations (1)–(3) implemented in the software DYNAMOF [45]. An iterative nonlinear least-squares algorithm [46] was employed to further minimize the error function. The “right” model was selected from χ^2^ analysis.

### 4.4. Protein Unfolding

2D [^1^H,^15^N] HSQC were recorded on a Bruker AVANCE III 600 MHz spectrometer, at 3 temperatures (10, 20, and 30 °C) and 15 different hydrostatic pressures (1,50, 100, 300, 500, 700, 900, 1100, 1300, 1500, 1700, 1900, 2100, 2300, and 2500 bar) for pressure denaturation, and at 20 °C and 13 different urea concentrations (0, 0.1, 0.2, 0.375, 0.75, 1.125, 1.5, 1.875, 2.25, 3, 3.75, 5, and 6 M) for chemical denaturation. Samples with about 1 mM concentration of ^15^N-labeled proteins were used on conventional 3 mm NMR tubes (200 µL of sample volume) for chemical denaturation, or in 5 mm o.d. ceramic tubes (330 µL of sample volume) from Daedelus Innovations (Aston, PA, USA) for pressure denaturation. Hydrostatic pressure was applied to the sample directly within the magnet using the Xtreme Syringe Pump also from Daedelus Innovations. Samples with different urea concentrations were prepared about 10 h before recording the NMR experiments used for chemical denaturation studies, although each pressure jump was separated by a 2-h relaxation time, to allow the protein to reach full equilibrium. In the case of pressure denaturation, relaxation times for the folding/unfolding reactions were estimated from a series of 1D NMR experiments recorded after 200 bar P-Jump, following the increase of the resonance band corresponding to the methyl groups in the unfolded state of the protein.

The cross-peak intensities for the folded species were measured at each pressure or each urea concentration, then fitted with a two-state model:

(4) $$I=\frac{I_{u}+I_{f}e^{-\left(\Delta G_{f}^{0}+p\Delta V_{f}^{0}\right)/RT}}{1+\;e^{-\left(\Delta G_{f}^{0}+p\Delta V_{f}^{0}\right)/RT}}$$

in the case of pressure denaturation, or:

(5) $$I=\frac{I_{u}+I_{f}e^{-\left(\Delta G_{f\;}^{0}+\;m\left[Urea\right]\right)/RT}}{1+\;e^{-\left(\Delta G_{f}^{0}\;+\;m\left[Urea\right]\right)/RT}}$$

in the case of chemical denaturation. In these equations, *I* is the cross-peak intensity measured at a given pressure or at a given urea concentration, and *I_f_* and *I_u_* correspond to the cross-peak intensities in the folded state (1 bar or 0 M urea) and in the unfolded state (2500 bar or 6 M urea), respectively. $\Delta G_{f}^{0}$ stands for the residue-specific apparent free energy at atmospheric pressure or at 0 M urea. $\Delta V_{f}^{0}$ corresponds to the residue-specific apparent volume of folding for pressure denaturation, while m is related to the steepness of the unfolding transition for chemical denaturation.

Native contact maps were obtained by using software CMView (http://www.bioinformatics.org/cmview/; accessed on 25 March 2020) with a threshold of 9 Å around the Cα of each residue, using the best structure obtained for GIPC1-GH2 among the 20 refined ones.

## 5. Conclusions

We demonstrate that combining NMR spectroscopy, which can bring information at an atomic resolution, with a mild and reversible method, such as high hydrostatic pressure, for protein unfolding can bring unprecedented details on the folding landscape of a protein. Here, we applied high-pressure NMR spectroscopy to the study of the folding/unfolding pathways of an α-helical bundle. Indeed, whereas similar studies have been widely applied to all-β or mixed-α/β 3D scaffolds, they are lacking for all-α helical structures. Unexpectedly, we found that the secondary and tertiary structures unfold simultaneously, although partial unfolding can occur. Importantly, this partial unfolding cannot be revealed with chemical denaturation, confirming the superiority of high pressure for exploring the folding landscape of a protein. Of course, whether these results obtained for GIPC1-GH2 can be generalized to other comparable α-helical bundles or whether more will have to be done remains an open question.

## Data Availability

Data is contained within the article or Supplementary Materials.

## Supplementary Materials

The following are available online at https://www.mdpi.com/article/10.3390/ijms22073597/s1: Table S1: NMR restraints and refinement statistics for GIPC1-GH2 solution structures, Figure S1: Crystal structure of the full-length GIPC1 protein, Figure S2: Phase modulated CLEANEX-PM experiment recorded on 15N-labeled GIPC1-GH2, Figure S3: Relaxation measurements performed on 15N-labeled GIPC1-GH2., Figure S4: Distribution of the values of the thermodynamic parameters ∆G^0^ and ∆V^0^, Figure S5: Local pressure stability of GIPC1-GH2, Figure S6: Local chemical stability of GIPC1-GH2. Figure S7: Estimation of the folded/unfolded fraction of GIPC1-GH2.

## Abbreviations and Nomenclature

GIPC1	GAIP Interacting Protein, C-terminus 1
GH2	C-terminal GIPC-homology 2
H(H)P-NMR	High (Hydrostatic) Pressure Nuclear Magnetic Resonance
nOe	nuclear Overhauser enhancement

## References

[1] Malhotra P., Udgaonkar J.B. (2016). How cooperative are protein folding and unfolding transitions?. Protein Sci..

[2] Sosnick T.R., Barrick D. (2011). The folding of single domain proteins—Have we reached a consensus?. Curr. Opin. Struct. Biol..

[3] Roche J., Royer C.A., Roumestand C. (2015). Exploring the Protein Folding Pathway with High-Pressure NMR: Steady-State and Kinetics Studies. High Press. Biosci..

[4] Roche J., Royer C.A., Roumestand C. (2017). Monitoring protein folding through high pressure NMR spectroscopy. Prog. Nucl. Magn. Reason. Spectrosc..

[5] Roche J., Royer C.A., Roumestand C. (2019). Exploring Protein Conformational Landscapes Using High-Pressure NMR. Methods Enzymol..

[6] Dubois C., Herrada I., Barthe P., Roumestand C. (2020). Combining High-Pressure Perturbation with NMR Spectroscopy for a Structural and Dynamical Characterization of Protein Folding Pathways. Molecules.

[7] Rouget J., Aksel T., Roche J., Saldana J., Garcia A.E., Barrick D., Royer C.A. (2011). Size and sequence and the volume change of protein folding. J. Am. Chem. Soc..

[8] Roche J., Caro J.A., Norberto D.R., Barthe P., Roumestand C., Schlessman J.L., Garcia A.E., García-Moreno B.E., Royer C.A. (2012). Cavities determine the pressure unfolding of proteins. Proc. Natl. Acad. Sci. USA.

[9] Roche J., Dellarole M.J., Caro A., Guca E., Norberto D.R., Yang Y.-S., Garcia A.E., Roumestand C., García-Moreno B., Royer C.A. (2012). Remodeling of the folding free-energy landscape of staphylococcal nuclease by cavity-creating mutations. Biochemistry.

[10] Herrada I., Barthe P., Vanheusden M., DeGuillen K., Mammri L., Delbecq S., Rico F., Roumestand C. (2018). Monitoring Unfolding of Titin I27 Single and Bi Domain with High-Pressure NMR Spectroscopy. Biophys. J..

[11] Saotome T., Doret M., Kulkarni M., Yang Y.-S., Barthe P., Kuroda Y., Roumestand C. (2019). Folding of the Ig-Like Domain of the Dengue Virus Envelope Protein Analyzed by High-Hydrostatic-Pressure NMR at a Residue-Level Resolution. Biomolecules.

[12] Fossat M.J., Dao T.P., Jenkins K., Dellarole M., Yang Y.-S., McCallum S.A., Garcia A.E., Barrick D., Roumestand C., Royer C.A. (2016). High-Resolution Mapping of a Repeat Protein Folding Free Energy Landscape. Biophys. J..

[13] Zhang S., Zhang Y., Stenzoski N.E., Zou J., Peran I., McCallum S.A., Raleigh D.P., Royer C.A. (2019). Pressure-Temperature Analysis of the Stability of the CTL9 Domain Reveals Hidden Intermediates. Biophys. J..

[14] Kohn W.D., Mant C.T., Hodges R.S. (1997). α-Helical protein assembly motifs. J. Biol. Chem..

[15] Katoh M. (2013). Functional proteomics, human genetics and cancer biology of GIPC family members. Exp. Mol. Med..

[16] Shang G., Brautigam C.A., Chen R., Lu D., Torres-Vázquez J., Zhang X. (2017). Structure analyses reveal a regulated oligomerization mechanism of the PlexinD1/GIPC/myosin VI complex. eLife.

[17] Shen Y., Bax A. (2013). Protein backbone ans side chain torsion angles predicted from NMR chemical shifts using artificial neural networks. J. Biomol. NMR.

[18] Güntert P. (2004). Automated NMR structure calculation with CYANA. Methods Mol. Biol..

[19] Hwang T.L., Mori S., van Zijl P.C. (1997). Application of phase-modulated CLEAN chemical EXchange spectroscopy(CLEANEX-PM) to detect water-protein proton exchange and intermolecular NOEs. J. Am. Chem. Soc..

[20] Hwang T.L., van Zijl P.C., Mori S. (1998). Accurate quantitation of water-amide proton exchange rates using the phase-modulated CLEAN chemical EXchange (CLEANEX-PM) approach with a Fast-HSQC (FHSQC) detection scheme. J. Biomol. NMR.

[21] Abragam A. (1961). Principles of Nuclear Magnetism.

[22] Lipari G., Szabo A. (1982). Model-free approach to the interpretation of nuclear magnetic resonance relaxation in macromolecules. J. Am. Chem. Soc..

[23] Clore G.M., Driscoll P.C., Wingfield P.T., Gronenborn A.M. (1990). Analysis of the backbone dynamics of interleukin-1 beta using two-dimensional inverse detected heteronuclear 15N-1H NMR spectroscopy. Biochemistry.

[24] Seemann H., Winter R., Royer C.A. (2001). Volume, expansivity and isothermal compressibility changes associated with temperature and pressure unfolding of Staphylococcal nuclease. J. Mol. Biol..

[25] Kitahara R., Royer C.A., Yamada H., Boyer M., Saldana J.L., Akasaka K., Roumestand C. (2002). Equilibrium and pressure-jump relaxation studies of the conformational transitions of P13MTCP1. J. Mol. Biol..

[26] Baase W.A., Liu L., Tronrud D.E., Matthews B.W. (2010). Lessons from the lysozyme of phage T4. Protein Sci..

[27] Pace C.N., Fu H., Fryar K.L., Landua J., Trevino S.R., Shirley B.A., Hendricks M.M., Iimura S., Gajiwala K., Scholtz J.M. (2011). Contribution of hydrophobic interactions to protein stability. J. Mol. Biol..

[28] Shortle D. (1995). Staphylococcal nuclease: A showcase of m-value effects. Adv. Protein Chem..

[29] Pons J.L., Malliavin T.E., Delsuc M.A. (1996). Gifa V.4: A complete package for NMR data set processing. J. Biomol. NMR.

[30] Sattler M., Schleucher J., Griesinger C. (1999). Heteronuclear multi-dimensional NMR experiments for the structure determination of proteins in solution employing pulsed field gradients. Prog. Nucl. Magn. Reson. Spectrosc..

[31] Nederveen A.J., Doreleijers J.F., Vranken W., Miller Z., Spronk C.A., Nabuurs S.B., Güntert P., Livny M., Markley J.L., Nilges M. (2005). RECOORD: A recalculated coordinate database of 500+ proteins from the PDB using restraints from the BioMagResBank. Proteins.

[32] Laskowski R.A., Moss D.S., Thornton J.M. (1993). Main-chain bond lengths and bond angles in protein structures. J. Mol. Biol..

[33] Koradi R., Billeter M., Wüthrich K. (1996). MOLMOL: A program for display and analysis of macromolecular structures. J. Mol. Graph..

[34] Delano W.L. (2002). PyMOL: An open-source molecular graphics tool. CCP4 Newslett. Protein Crystallogr..

[35] Peng J.W., Wagner G. (1992). Mapping of the spectral densities of N-H bond motion in eglin c using heteronuclear relaxation experiments. Biochemistry.

[36] Peng J.W., Wagner G. (1992). Mapping of the spectral density functions using heteronuclear NMR relaxation experiments. J. Magn. Reson..

[37] Kay L.E., Nicholson L.K., Delaglio F., Bax A., Torchia D.A. (1992). Pulse sequences for removal of the effects of cross correlation between dipolar and chemical-shift anisotropy relaxation mechanisms on the measurement of heteronuclear T1 and T2 values in proteins. J. Magn. Reson..

[38] Barthe P., Chiche L., Declerck N., Delsuc M.A., Lefèvre J.F., Malliavin T., Mispelter J., Stern M.-H., Lhoste J.M., Roumestand C. (1999). Refined Solution Structure and Backbone Dynamics of 15N-Labeled C12A-p8MTCP1 Studied by NMR Relaxation. J. Biomol. NMR.

[39] Guignard L., Padilla A., Mispelter J., Yang Y.-S., Stern M.-H., Lhoste J.M., Roumestand C. (2000). Backbone dynamics and solution structure refinement of the 15N-labeled human oncogenic protein p13MTCP1: Comparison with X-ray data. J. Biomol. NMR.

[40] Auguin D., Barthe P., Augé-Sénégas M.T., Stern M.H., Noguchi M., Roumestand C. (2004). Solution structure and backbone dynamics of the pleckstrin homology domain of the human protein kinase B (PKB/Akt). Interaction with inositol phosphates. J. Biomol. NMR.

[41] Farrow N.A., Zhang O., Szabo A., Torchia D.A., Kay L.E. (1995). Spectral density function mapping using 15N relaxation data exclusively. J. Biomol. NMR.

[42] Ishima R., Nagayama K. (1995). Protein backbone dynamics revealed by quasi spectral density function analysis of amide N-15 nuclei. Biochemistry.

[43] Ishima R., Nagayama K. (1995). Quasi-spectral-density function analysis for nitrogen-15 nuclei in proteins. J. Magn. Reson. B.

[44] Lefèvre J.-F., Dayie K.T., Peng J.W., Wagner G. (1996). Internal mobility in the partially folded DNA binding and dimerization domains of GAL4: NMR analysis of the N-H spectral density functions. Biochemistry.

[45] Barthe P., Ropars V., Roumestand C. (2006). DYNAMOF: A program for the dynamics analysis of relaxation data obtained at multiple magnetic fields. C. R. Chimie.

[46] Press W.H., Flannery B.P., Teukolsky S.A., Vetterling W.T. (1986). Numerical Recipes.

