# Supplementary material for: Pressure and Chemical Unfolding of an α-Helical Bundle Protein: The GH2 Domain of the Protein Adaptor GIPC1
Source: Int J Mol Sci. 2021 Mar 30;22(7):3597. doi: 10.3390/ijms22073597 (PMC8037465; doi:10.3390/ijms22073597)
Supplement: Supplementary file 1 [file ijms-22-03597-s001.zip › SupplementaryMaterials_Rev/Figure S1.docx]

**Supplementary Material, Figure S1**

**
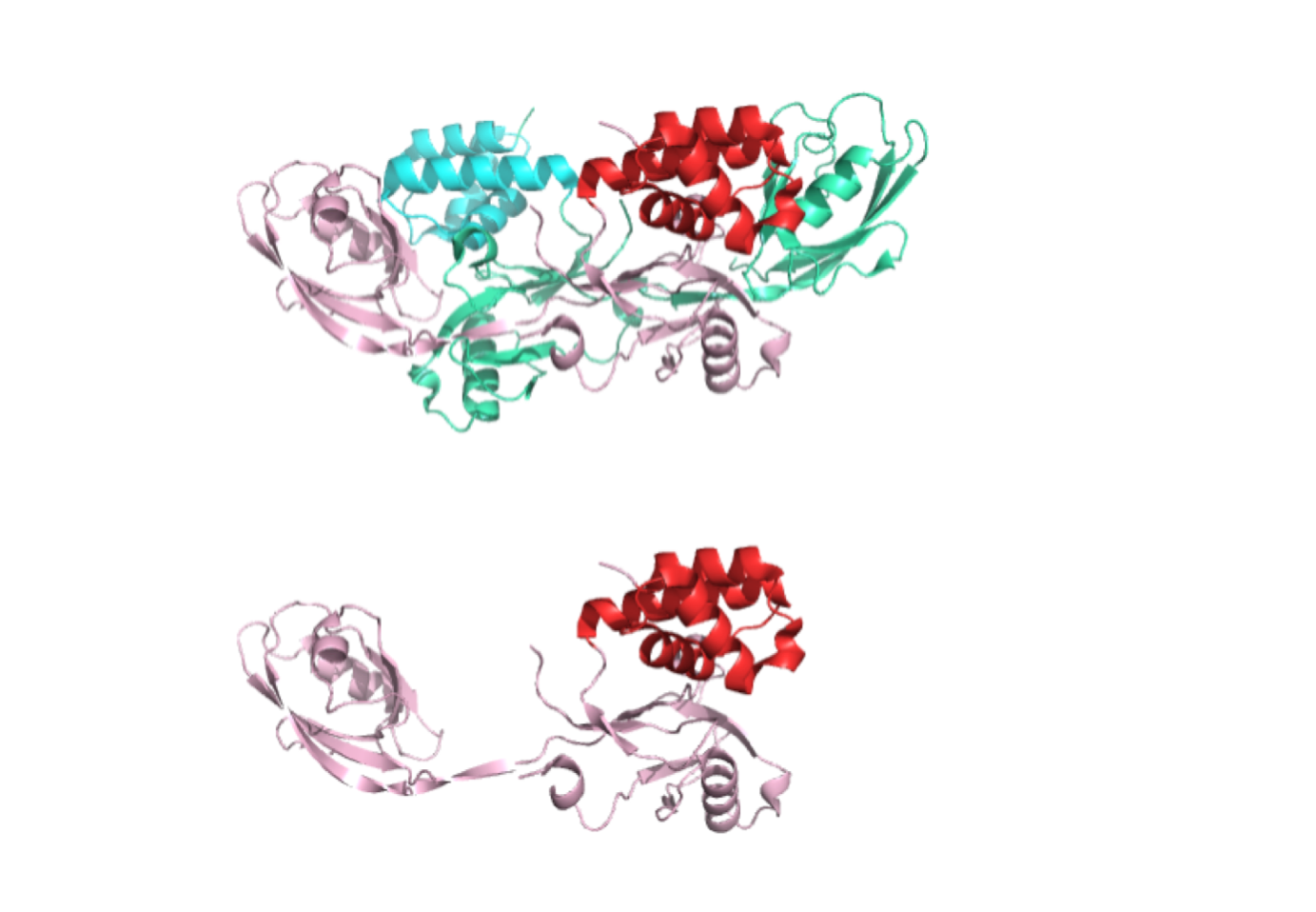
**

**Figure S1.** Crystal structure of the full-lengh GIPC1 protein [Shang et al., 2017; PDB code: 5V6b]. (Top) GIPC1 is a multi-domain dimeric protein (Top view: one monomer in light blue, with the GH2 domain highlighted in cyan, the other monomer in pink, with the GH2 domain highlighted in red). (Bottom) Each monomer comprises a GH1 N-terminal domain that adopts an ubiquitin-like fold, followed by a PDZ domain, and the GH2 domain (residues 255-333) forming a four-helix bundle (displayed in red in the figure).

Shang G, Brautigam CA, Chen R, Lu D, Torres-Vázquez J, Zhang X. Structure analyses reveal a regulated oligomerization mechanism of the PlexinD1/GIPC/myosin VI complex. Elife. 2017 May 24;6:e27322. doi: 10.7554/eLife.27322.
