# Supplementary material for: Pressure and Chemical Unfolding of an α-Helical Bundle Protein: The GH2 Domain of the Protein Adaptor GIPC1
Source: Int J Mol Sci. 2021 Mar 30;22(7):3597. doi: 10.3390/ijms22073597 (PMC8037465; doi:10.3390/ijms22073597)
Supplement: Supplementary file 1 [file ijms-22-03597-s001.zip › SupplementaryMaterials_Rev/Figure S2.docx]

**Supplementary Material, Figure S2**

**
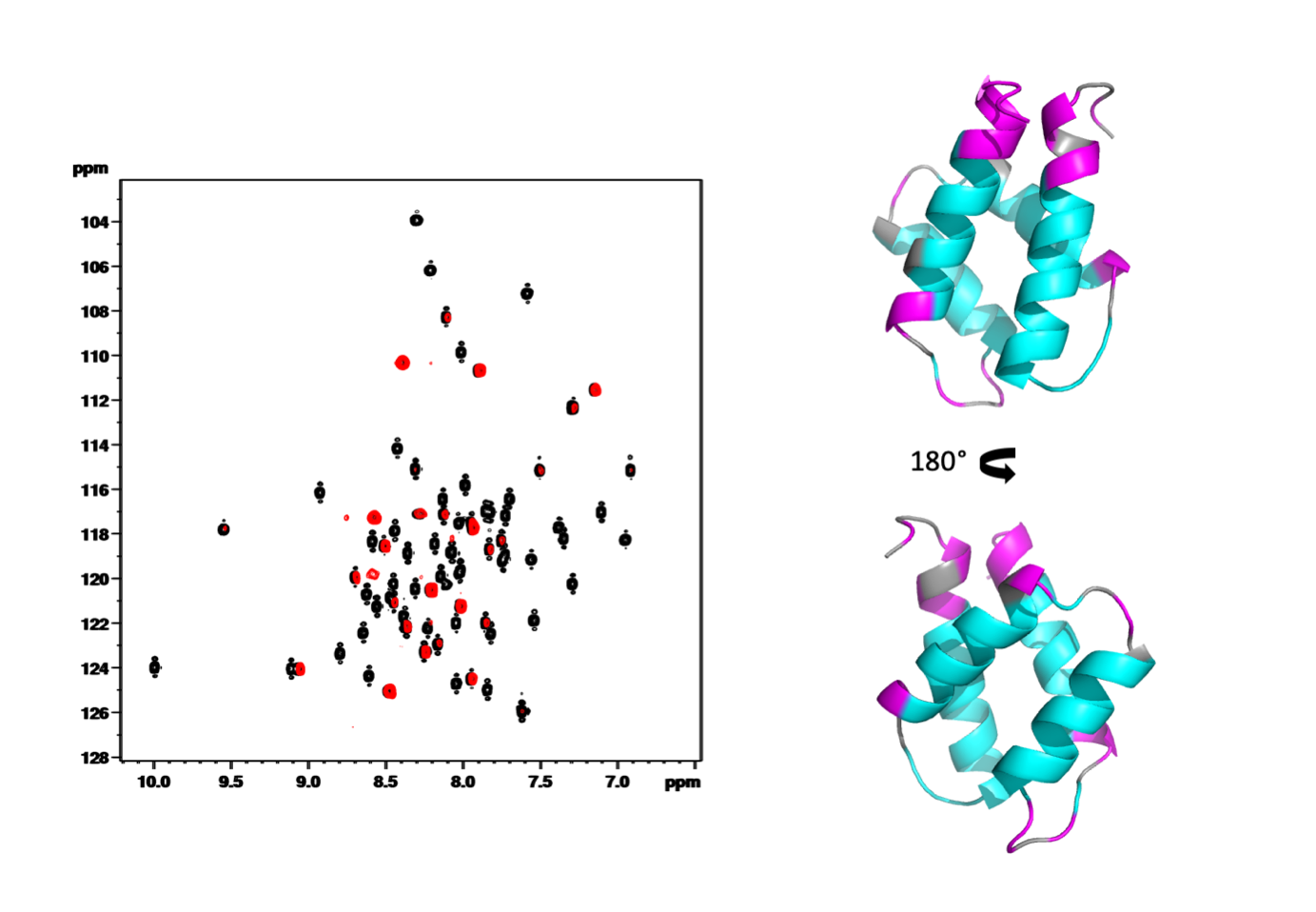
**

**Figure S2.** Phase modulated CLEANEX-PM experiment [Hwang et al., 1997, 1998] recorded on ^15^N-labeled GIPC1-GH2. (Left) Superimposition of a clean chemical exchange experiment (CLEANEX) recorded with 100 ms mixing time (red) and a reference HSQC spectra (black). Experiments were recorded with the same experimental conditions as in Figure 1. (Right) The residues exhibiting amide cross-peaks in the CLEANEX experiments were coloured in pink on the cartoon representation of the 3D solution structure of GIPC1-GH2 (two views rotated by 180°C). The corresponding amide protons are supposed to be solvent exposed and not involved in intra-molecular H-bonds. For CYANA modeling of the 3D structure, H-bonds restraints where introduced between amide donnors and carbonyle acceptors when the corresponding amide HSQC cross-peaks did not overlap with amide CLEANEX cross-peak, and when the corresponding residue was involved in a regular helical structure, as deduced from TALOS analysis.

Hwang, T. L., S. Mori, ., P. C. M. van Zijl. 1997. Application of phase-modulated CLEAN chemical EXchange spectroscopy

(CLEANEX-PM) to detect water-protein proton exchange and intermolecular NOEs. J. Am. Chem. Soc. 119:6203–6204.

Hwang, T. L., P. C. van Zijl, and S. Mori. 1998. Accurate quantitation of water-amide proton exchange rates using the phase-modulated CLEAN chemical EXchange (CLEANEX-PM) approach with a Fast-HSQC (FHSQC) detection scheme. J. Biomol. NMR. 11:221–226.
