# Supplementary material for: Pressure and Chemical Unfolding of an α-Helical Bundle Protein: The GH2 Domain of the Protein Adaptor GIPC1
Source: Int J Mol Sci. 2021 Mar 30;22(7):3597. doi: 10.3390/ijms22073597 (PMC8037465; doi:10.3390/ijms22073597)
Supplement: Supplementary file 1 [file ijms-22-03597-s001.zip › SupplementaryMaterials_Rev/Figure S3.docx]

**Supplementary Material, Figure S3**

**
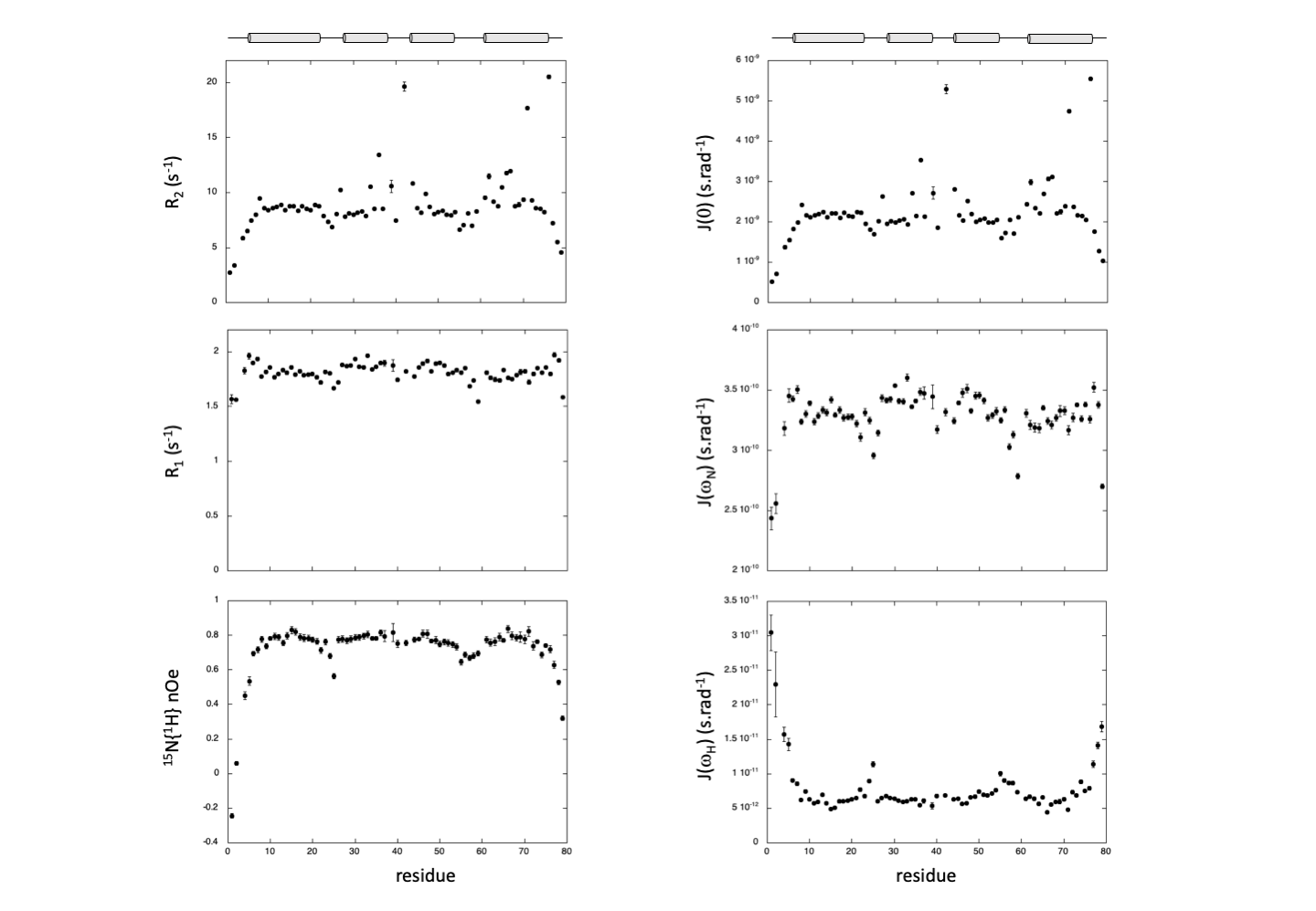
**

**Figure S3.** Relaxation measurements performed on 15N-labeled GIPC1-GH2. (Left) From top to bottom: 15N R2, R1 heteronuclear relaxation rates and [1H,15N] nOes are displayed versus the protein sequence. (Right) From top to bottom: J(0), J(wN) and <J(wH)> spectral densities values displayed versus the sequence. This values where obtained according Equation X, from the values of the corresponding relaxation parameters (see Materials and Methods). The location of the four helices in the protein sequence is schematized with cylinders on top of the figure.
