# Supplementary material for: Pressure and Chemical Unfolding of an α-Helical Bundle Protein: The GH2 Domain of the Protein Adaptor GIPC1
Source: Int J Mol Sci. 2021 Mar 30;22(7):3597. doi: 10.3390/ijms22073597 (PMC8037465; doi:10.3390/ijms22073597)
Supplement: Supplementary file 1 [file ijms-22-03597-s001.zip › SupplementaryMaterials_Rev/Figure S4.docx]

**Supplementary Material, Figure S4**

**
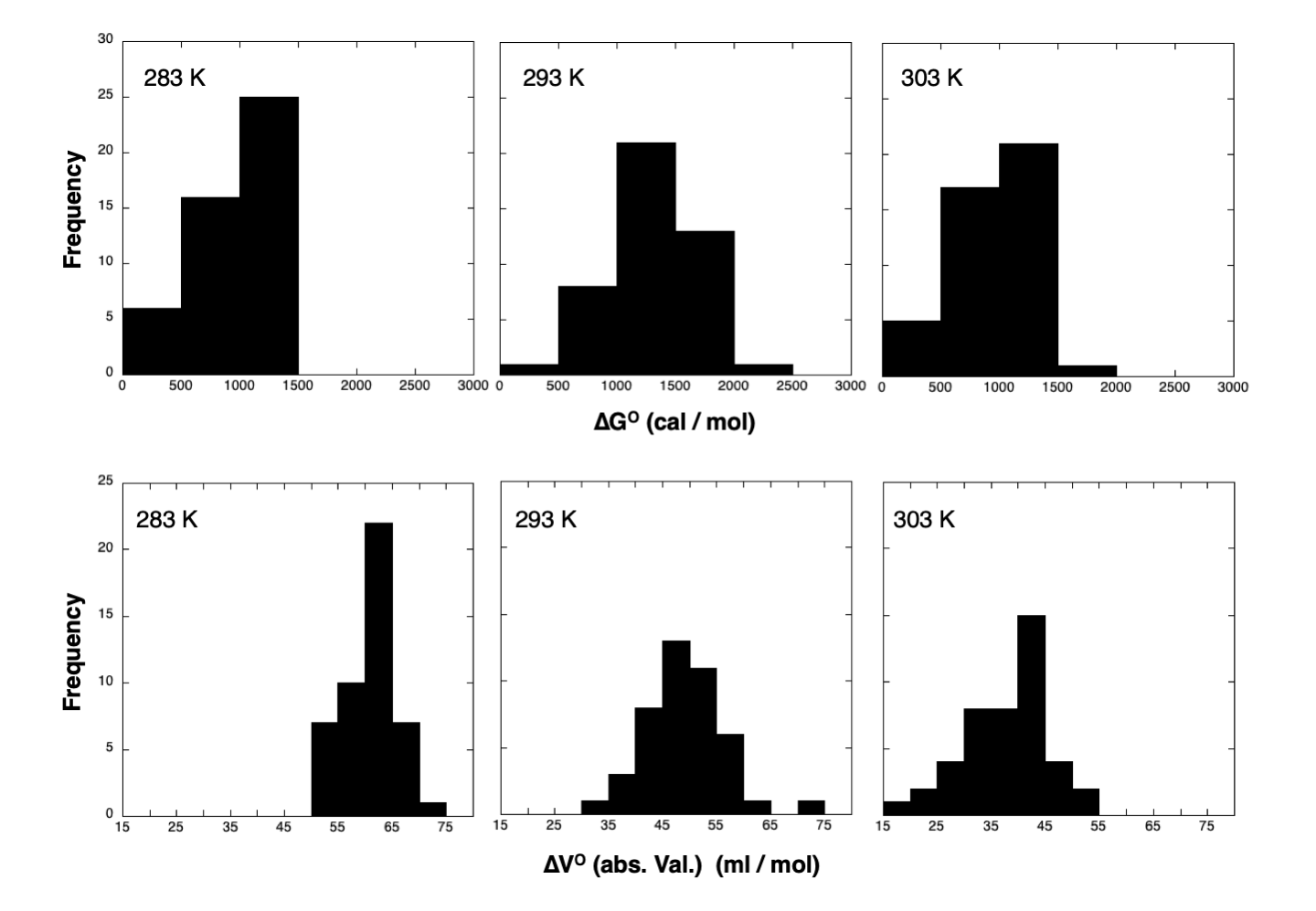
**

**Figure S4.** Distribution of the values of ${\Delta G}_{u}^{0}$ (top) and ${\Delta V}_{u}^{0}$ (bottom) at 283 K, 293 K and 303 K (from left to right).
