# Supplementary material for: Pressure and Chemical Unfolding of an α-Helical Bundle Protein: The GH2 Domain of the Protein Adaptor GIPC1
Source: Int J Mol Sci. 2021 Mar 30;22(7):3597. doi: 10.3390/ijms22073597 (PMC8037465; doi:10.3390/ijms22073597)
Supplement: Supplementary file 1 [file ijms-22-03597-s001.zip › SupplementaryMaterials_Rev/Figure S5.docx]

**Supplementary Material, Figure S5**

**
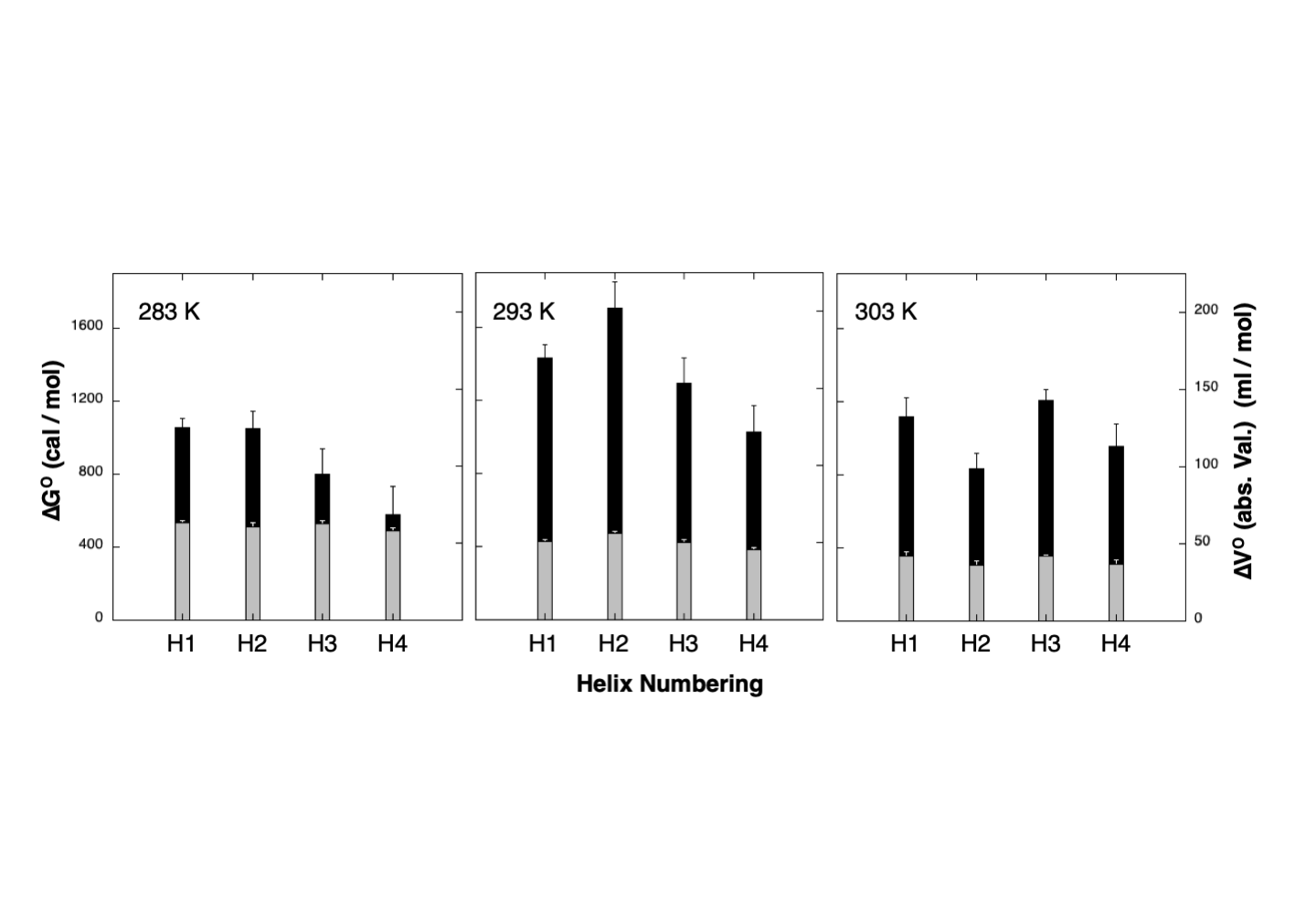
**

**Figure S5. Local pressure stability of GIPC1-GH2** at 283, 293 and 303K, as indicated. The average values of ${\Delta G}_{u}^{0}$ (black bars) and ${\Delta V}_{u}^{0}$ (dashed bars) are reported for each helix.
