# Supplementary material for: Pressure and Chemical Unfolding of an α-Helical Bundle Protein: The GH2 Domain of the Protein Adaptor GIPC1
Source: Int J Mol Sci. 2021 Mar 30;22(7):3597. doi: 10.3390/ijms22073597 (PMC8037465; doi:10.3390/ijms22073597)
Supplement: Supplementary file 1 [file ijms-22-03597-s001.zip › SupplementaryMaterials_Rev/Figure S6.docx]

**Supplementary Material, Figure S6**

**
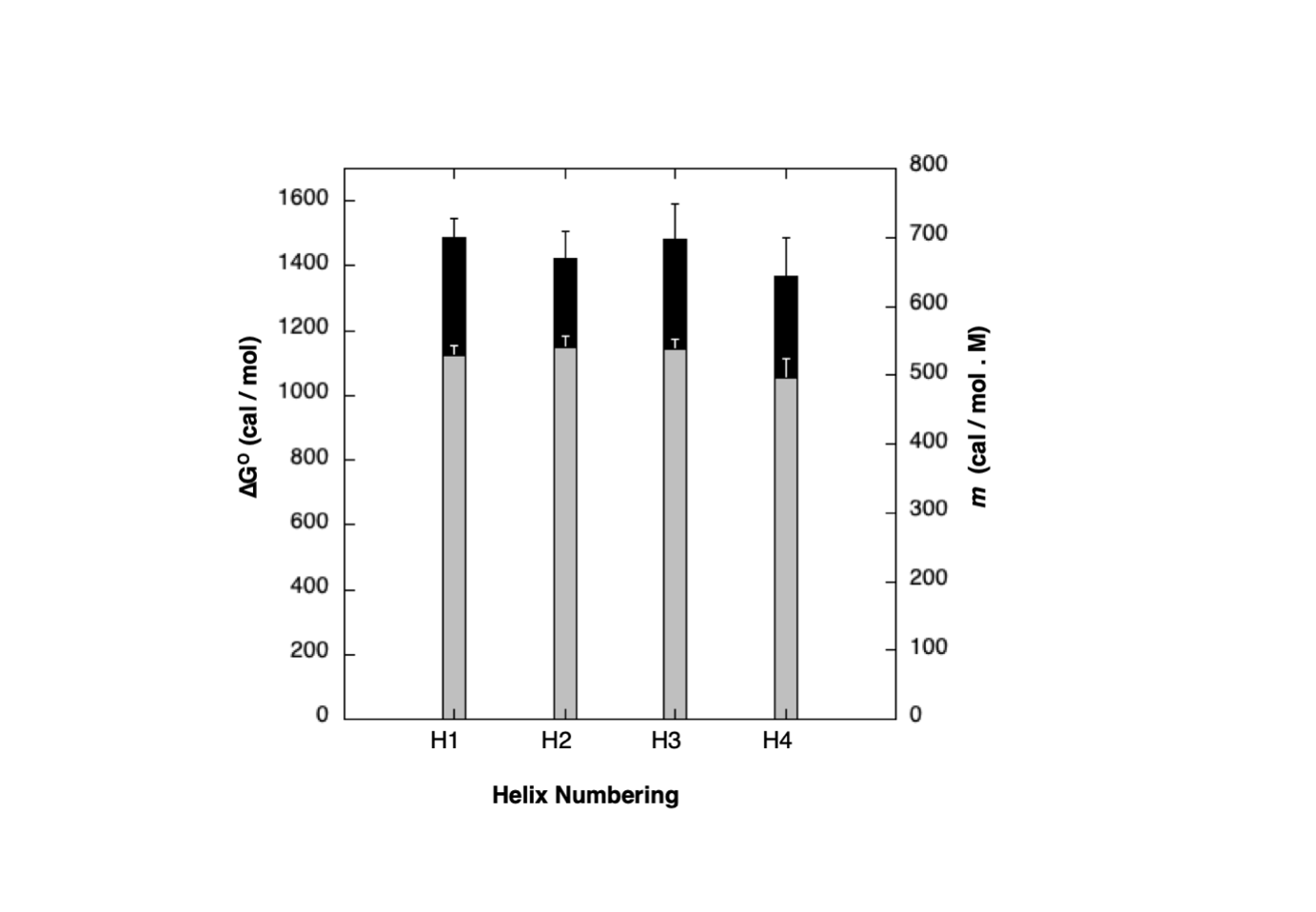
**

**Figure S6. Local chemical stability of GIPC1-GH2** at 293K. The average ${\Delta G}_{u}^{0}$ (black bars) and m (dashed bars) values of are reported for each helix. The values measured for each helix fall within experimental errors.
