# Supplementary material for: Pressure and Chemical Unfolding of an α-Helical Bundle Protein: The GH2 Domain of the Protein Adaptor GIPC1
Source: Int J Mol Sci. 2021 Mar 30;22(7):3597. doi: 10.3390/ijms22073597 (PMC8037465; doi:10.3390/ijms22073597)
Supplement: Supplementary file 1 [file ijms-22-03597-s001.zip › SupplementaryMaterials_Rev/Figure S7.docx]

**Supplementary Material, Figure S7**

**
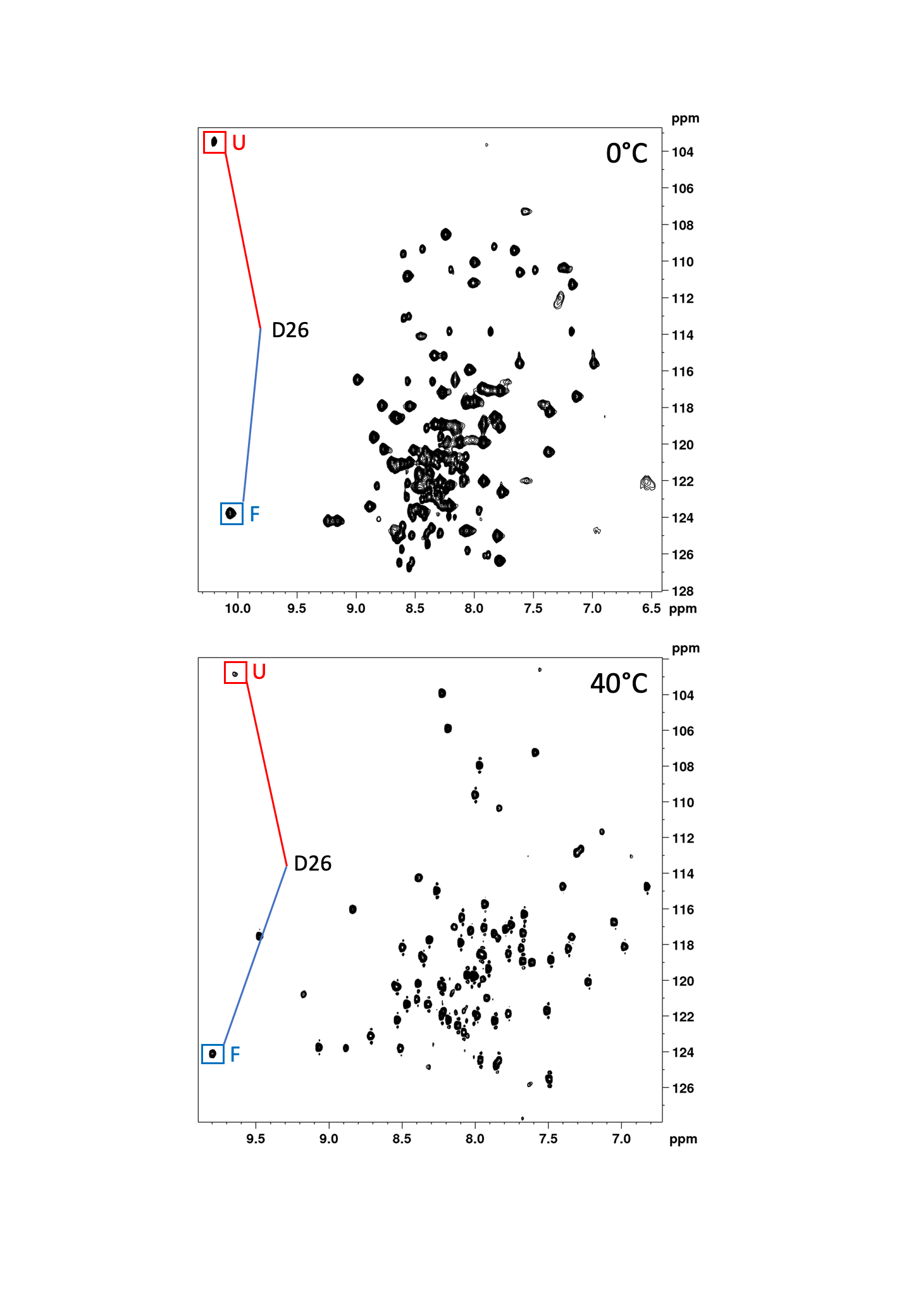
**

**Figure S7. Estimation of the Folded/Unfolded fraction of GIPC1-GH2.** For each HSQC recorded at ambient pressure and at 0°C (top) and 40°C (bottom), the volumes of the the D26 amide cross-peaks corresponding to the folded (F, blue square) and unfolded (U, red square) species were measured to estimate the folded fraction ( ƒ_(F)_ ) of GIPC20 through the relation: ƒ_(F)_ = F/(U+F). Note that the cross-peak corresponding to the unfolded species is aliased along the ^15^N dimension.
