# Supplementary material for: Pressure and Chemical Unfolding of an α-Helical Bundle Protein: The GH2 Domain of the Protein Adaptor GIPC1
Source: Int J Mol Sci. 2021 Mar 30;22(7):3597. doi: 10.3390/ijms22073597 (PMC8037465; doi:10.3390/ijms22073597)
Supplement: Supplementary file 1 [file ijms-22-03597-s001.zip › SupplementaryMaterials_Rev/Table S1.docx]

**Supplementary Material, Table S1**

**Table S1:** NMR restraints and refinement statistics for GIPC1-GH2 solution structures

|  | **Protein** |
| --- | --- |
| **NMR distance and dihedral constraints** |  |
| Distance constraints |  |
| Total NOE | 1219 |
| Intra-residue | 281 |
| Inter-residue |  |
| Sequential (\|*i* – *j*\| = 1) | 352 |
| Medium-range (\|*i* – *j*\| < 4) | 349 |
| Long-range (\|*i* – *j*\| > 5) | 237 |
| Hydrogen bonds | 88 |
| Total dihedral angle restraints |  |
| φ | 69 |
| ψ | 69 |
| χ_1_ | 26 |
|  |  |
| **Structure statistics** |  |
| Violations (mean and s.d.) |  |
| Max. distance constraint violation (Å) | 0.13 ± 0.02 |
| Max. dihedral angle violation (º) | 1.17 ± 0.53 |
| Deviations from idealized geometry |  |
| Bond lengths (Å) | 0.0090 ± 0.0003 |
| Bond angles (º) | 1.0888 ± 0.0469 |
| Impropers (º) | 1.2349 ± 0.0650 |
|  |  |
| **Ramachandran plot (%)** |  |
| Most favoured region | 95.6 |
| Additionally allowed region | 4.4 |
| Generously allowed region | 0.0 |
| Disallowed region | 0.0 |
|  |  |
| **Average pairwise** *r.m.s.* **deviation** (Å)** |  |
| Backbone | 0.47 ± 0.09 |
| Heavy | 1.14 ± 0.14 |

** “Pairwise r.m.s. deviation calculated among 20 refined structures for residues 5-76.”
